# Supplementary material for: Generation of C9orf72 repeat knock-in iPSC lines for modelling ALS and FTD
Source: bioRxiv. 2025 Feb 11:2025.02.10.637041. Preprint. [Version 1] doi: 10.1101/2025.02.10.637041 (PMC12478360; doi:10.1101/2025.02.10.637041)
Supplement: 1 [file NIHPP2025.02.10.637041v1-supplement-1.pdf]

bioRxiv preprint doi: https://doi.org/10.1101/2025.02.10.637041; this version posted February 11, 2025. The copyright holder for this preprint (which was not certified by peer review) is the author/funder, who has granted bioRxiv a license to display the preprint in perpetuity. It is made available under aCC-BY 4.0 International license.

Supplementary Figures

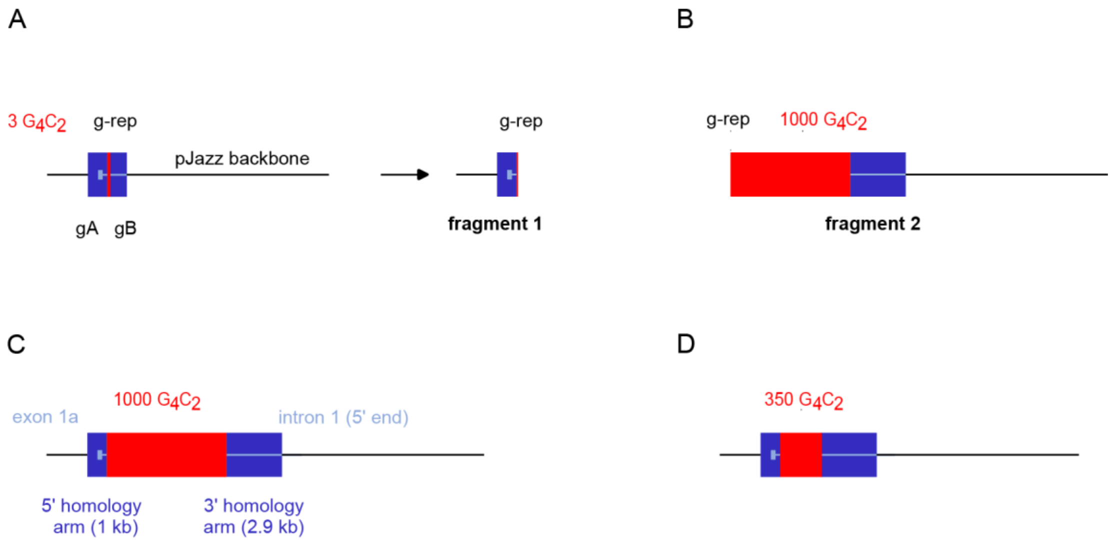

Supplemental Figure 1. Assembly of the human iPSC targeting construct. (**A**) A ~2kb fragment of human *C9orf72* including the G_4C_2 region with 3 repeats was first isolated via CRISPR-Cas9 digestion from a human C9orf72 locus BAC clone (RP11-27J8), using sgRNA guides A and B (gA and gB), and blunt end cloned into the pJazz-OK backbone. CRISPR-Cas9 digestion with an sgRNA guide targeting within the 5' end of the repeat sequence (g-rep) was then used to generate fragment 1, constituting the 5' homology arm (~1kb) together with the short arm of the pJazz vector. (**B**) Fragment 2 was derived from an existing construct originally designed for generating mouse models (from 'pJazz-V4' [10]), and harbours 1000 G_4C_2 repeats plus 2.9 kb of flanking human sequence (3' homology arm), together with the long arm of the pJazz vector; and was isolated from its parent vector via CRISPR-Cas9 digestion with sgRNA guide g-rep, cutting within the 5' end of the long repeat. (**C**) Fragments 1 and 2 were ligated together to form a targeting construct with a seamless 1000 G_4C_2 repeat expansion within the endogenous human genomic sequence. (**D**) A natural retraction of the repeat during the final cloning step additionally yielded a 350-repeat targeting construct. The 350-repeat targeting construct was taken forward, as it was non-toxic, in contrast to the 1000-repeat construct, which resulted in cell death following nucleofection. sgRNA gA: AACGTTTTAATCATTCACCG; sgRNA gB: TTTCTGAATACAAAGCCTGG; sgRNA g-rep: AGGAGTCGCGCGCTAGGGGC.

bioRxiv preprint doi: https://doi.org/10.1101/2025.02.10.637041; this version posted February 11, 2025. The copyright holder for this preprint (which was not certified by peer review) is the author/funder, who has granted bioRxiv a license to display the preprint in perpetuity. It is made available under aCC-BY 4.0 International license.

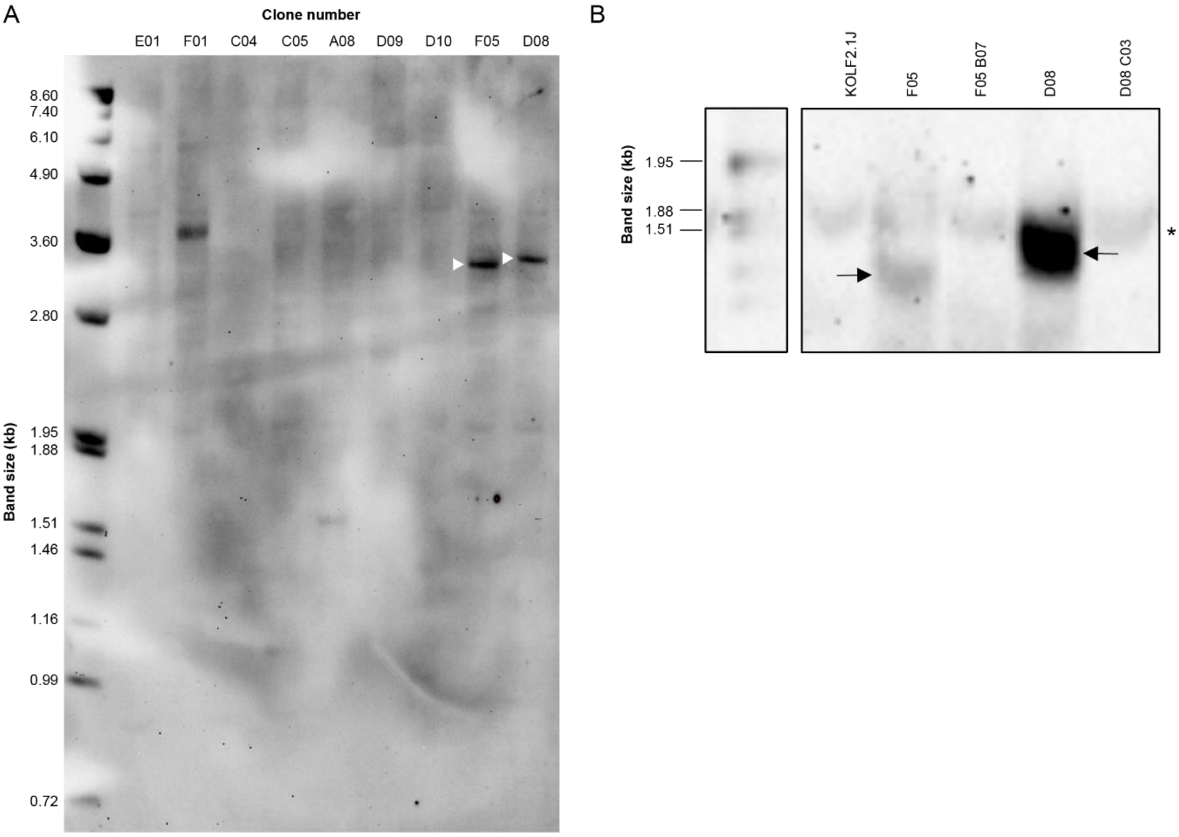

Supplemental Figure 2. Southern blotting of repeat knock-in and revertant lines. (**A**) Southern blot of GGGGCC repeats in initial iPSC knock-in clones, demonstrating successful integration of expanded repeats in clones F05 and D08 (white arrows). (**B**) Southern blot of F05 and D08, with their respective revertant lines and the parental KOLF2.1J shows repeat expansions of approximately 200 repeats in F05 and D08 and successful removal in the revertant lines, *non-specific band.

bioRxiv preprint doi: https://doi.org/10.1101/2025.02.10.637041; this version posted February 11, 2025. The copyright holder for this preprint (which was not certified by peer review) is the author/funder, who has granted bioRxiv a license to display the preprint in perpetuity. It is made available under aCC-BY 4.0 International license.

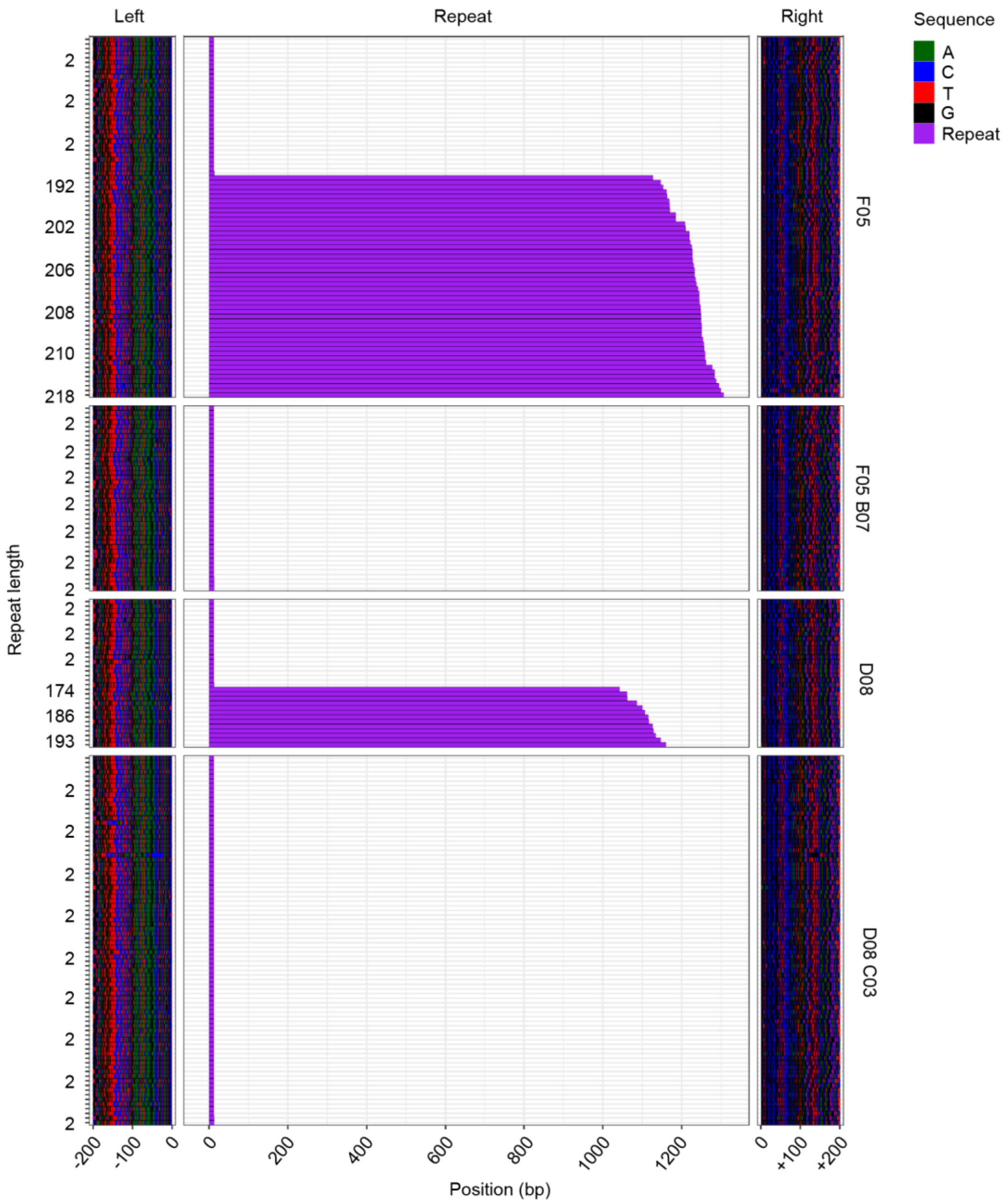

Supplemental Figure 3. Waterfall plots showing repeat length distribution and flanking sequence for each *C9orf72* repeat knock-in and revertant line. ONT long-read *C9orf72* sequences were trimmed to 200 bases flanking the hexanucleotide repeat. Each row represents a read, with the x-axis indicating base position along the read and the y-axis giving hexanucleotide repeat length. The plot is divided into three horizontal facets; *Left*, *Repeat* and *Right*, corresponding to the 200-base flanking regions and the repeat region. Coloured tiles represent individual nucleotide bases, with the colour scheme detailed in the key.
